# Temporal Resolution: performance of school-aged children in the GIN - Gaps-in-noise test

**DOI:** 10.1590/S1808-86942010000600013

**Published:** 2015-10-19

**Authors:** Maria Isabel Ramos do Amaral, Maria Francisca Colella-Santos

**Affiliations:** 1 Speech therapist, master's degree in Child and Adolescent Health, Center for Investigation in Pediatrics, CIPED/FCM/UNICAMP; 2 Speech therapist, doctoral degree in The Science of Human Communication Disorders, UNIFESP/EPM. Faculty member and coordinator of the Speech Therapy Course, Medical School (Faculdade de Ciências Médicas), UNICAMP

**Keywords:** hearing, child, auditory perception

## Abstract

**Abstract:**

Time resolution hearing skill is the minimum time necessary to solve acoustic events, which is fundamental for speech understanding, and which may be assessed by gap-detection tests, such as the Gaps-in-noise test (GIN).

**Aim:**

the purpose of this study was to verify the performance of time processing ability in children with no hearing and/or education difficulties by applying the GIN test in both genders and ages from 8 to 10 years.

**Study design:**

a prospective cross-sectional contemporary cohort.

**Material and method:**

The GIN test was applied to 75 school-aged children separated into three groups by age.

**Results:**

The findings showed no statistical differences among age groups or ears. Males had slightly better responses than females on the percentage of correct responses only.

**Conclusion:**

The gap threshold and percentage of correct responses were calculated regardless of the ear, gender or age, and were respectively 4.7ms and 73.6%. Based on a 95% confidence interval, the cut-off criterion for normal and abnormal performance was 6.1ms for the mean gap detection threshold and 60% for the percentage of correct responses.

## INTRODUCTION

The act of “listening” refers to more than merely detecting acoustic signals, as several neurophysiological and cognitive mechanisms and processes are needed for decoding, perceiving, recognizing and interpreting an auditory signal. The central auditory nervous system is highly complex and has built-in redundancy, since hearing has a relevant and essential role for correctly recognizing and discriminating auditory events, from the simplest signal such as a non-verbal stimulus to complex messages such as speech and language.^1^

Auditory processing is the term used to describe several mental operations to deal with auditory information; it depends on an innate biological ability, maturation, and acoustic sounds and experiences.^2^

Temporal auditory processing consists of perceiving sound and changes of this sound within a specific time period; it is part of most auditory processing abilities, as much auditory information is at least partially affected by time.^3^ It appears that temporal processing abilities are the basis of auditory processing, especially speech perception; acoustic frequency, intensity and time cues need to be processed precisely along the entire auditory system for the spoken message to be correctly decoded.^4^

Notwithstanding the complex relations between auditory processing, language, and learning disorders, many children with learning disabilities present changes in temporal auditory processing.^5^, ^6^, ^7^ Temporal resolution is an important factor in speech perception because it helps identify minor phonetic elements in discourse; changes in this auditory ability suggest interferences on normal speech perception and phoneme recognition.^8^, ^9^

Most patterns in speech sounds are based on millisecond time differences; thus, several tests of temporal resolution have been developed. These tests are based on the detection of time intervals between segments, the so-called gaps.^10^, ^11^

Musiek et al. developed the gaps-in-noise (GIN) test^12^ to assess gap-detection thresholds in clinical practice. This test includes parameters for temporal assessments such as the use of non-verbal material, gaps in white noise, and random gaps.^13^ Subjects are asked to answer if a gap is perceived; in every test list each gap will appear six times, totaling 60 gaps. This feature avoids “yes or no” responses only, increasing the reliability of the threshold.^12^

The GIN test is used for detecting altered temporal resolution in adults and children. Studies have attempted to relate speech and writing impairments in subjects with altered temporal resolution and without other conditions. The results have indicated that subjects with phonological deviation and/or impaired reading and writing may show changes in temporal auditory processing; these subjects may require more time for detecting time intervals between auditory stimuli compared to individuals without these changes.^14^, ^15^, ^16^

Central auditory assessment should evaluate subjects with auditory processing complaints, describe altered abilities, and guide phonological rehabilitation. In this context, studies of central auditory nervous system neural maturation are essential for standardizing the expected responses in behavioral tests; such studies establish criteria for each age group and each test.^17^

Samelli and Schochat^4^ proposed the Brazilian normatization of GIN test gap-detection thresholds in a population of normal-hearing adults. This evaluation included 100 adult subjects, 50 male and 50 female, ranging from age 18 to 30 years. The results showed no statistically significant differences among the four lists. The authors proposed applying two lists for evaluating temporal resolution, arguing that this would not alter the results, and would reduce the test time. The GIN test is reliable and adequate for the clinical routine. The authors also highlighted the need for normatizing the GIN test for subjects aged below 18 years; some international authors have stated that the temporal resolution test performance in children reaches adult levels at around ages 7 years,^18^ 9 years,^19^ 10 years,^20^ or 12 years.^23^ Not only these results diverge, but a Brazilian version of this test has not been validated in this age range.

Balen et al.^21^ recently studied temporal processing in 19 normally developing children using two temporal resolution tests, one of this was the GIN test. The results of the GIN test in 10 children aged 6 to 14 years revealed that the mean threshold was 5.7 ms (right ear) and 5.4 ms (left ear). There were differences between the two tests; the authors also emphasized the importance of assessing the temporal resolution in clinical auditory processing protocols and of establishing standards for the Brazilian child population.

Thus, the purpose of this study was to verify the performance of temporal resolution in children with no auditory complaints and/or difficulties at school by using the GIN test; the study included male and female subjects aged 8, 9, and 10 years.

## MATERIAL AND METHOD

A contemporary cohort prospective crosssectional study was carried out at our institution. The institutional review board approved this study (protocol no. 626/2007).

The sample comprised 75 school-aged children, of which 35 were female and 40 were male; the age ranged from 8 to 10 years. The children were enrolled in the basic education level of a public school in the city of Campinas. Subjects were allocated to three groups, as follows: 25 children aged 8 years comprised group I; 25 children aged 9 years comprised group II; and 25 children aged 10 years comprised group III.

The inclusion criteria were: age from 8 to 10 years, being a student in the public school network, not having difficulties at school as reported by the teacher in charge in a questionnaire on the school performance of each student, absence of complaints and/or hearing difficulties, normal results in a basic audiological assessment and the simplified evaluation of auditory processing. Children not in any of these criteria were excluded from the sample and referred to a complete otorhinolaryngological evaluation and treatment.

The children that were selected and their teachers were invited by letter and telephone to parents or caretakers, who signed a free informed consent form to include the subjects in the study. A medical history was taken; at this point, children that had undergone speech therapy for learning, reading or writing purposes, as well as children with a history of recurring otitis media and/or other conditions were excluded from the study.

The following procedures were carried out: meatoscopy, pure tone audiometry, logoaudiometry, immittance testing and a simplified evaluation of auditory processing that consisted of the localization of sounds test, the verbal and non-verbal sounds for sequential memory test (PSI/SSIMCC), and the dichotic digit test (binaural integration step).

The normal values in basic audiological evaluation were: auditory thresholds £ 15 dBHL at all tested frequencies (250 to 8,000 Hz),^22^ 88 to 100 % correct answers in the monosyllable list of the speech recognition index (SRI),^23^ a type A tympanometric curve, and the presence of ipsi- and contralateral reflexes from 70 to 100 dBHL at 500, 1,000, 2,000, 3,000 and 4,000 Hz.^24^ The normal range in the simplified auditory processing evaluation was correctly answering four or five directions in the localization of sounds test as long as the right and left directions had been indicated correctly; also, two or three sequences had to be correct in the verbal sequential memory and non-verbal sequential memory tests.^20^ Normal results for 8-year-old children in the dichotic digit test was a percentage equal to or higher than 85% in the right ear and 82% in the left ear; and a percentage of correct answers bilaterally equal to or higher than 95% in children aged 9 years or over.^25^

Children with normal results underwent the GIN test.^12^ An Interacoustics AC40 audiometer with a Philips compact disc recorder, in an acoustic booth, was used for this test; the intensity was 50 dBSL (according to the mean pure tone thresholds at 500, 1,000 and 2,000 Hz). The test was monaural. The compact disc contains a training track and four test tracks. Each test consists of several 6-second segments of white interspersed with 5-second silent segments. There are several gaps in different positions and variable duration within white noise segments. The gaps may last 2, 3, 4, 5, 6, 8, 10, 12, 15 or 20 ms; each one appears six times in each track, totaling 60 gaps per track. There are no gaps between a few segments. Patients are trained more than once with the training track until he or she understands the task. Half of the training track was applied to one ear and the other half to the opposite ear.

Two tracks (track 1 and track 2) were used so that fatigue of the child did not interfere with the results. The child was told that he or she would hear a noise and that there would be silent gaps; the child was to raise the hand whenever a gap was perceived. They were told that there would be not more than three gaps in any given noise segment, and that there would be segments without gaps. False-positives (child raises the hand without any gap occurring) were recorded; more than two false-positives were considered as errors.

The test presentation order was randomly alternated between the right and left ear; about half of the children started with track 1 on the right ear, and the remaining children started on the left ear. The gap-detection threshold (the shortest gap perceived in at least 66.6% of presentations - or four times out of six) and the percentage of correct answers per track (how many gaps were perceived in total) were calculated.

The SAS software version 9.1.3 was used for the statistical analysis. The significance level was 0.05 and was marked with an asterisk (*).

## RESULTS

**Chart 1:**
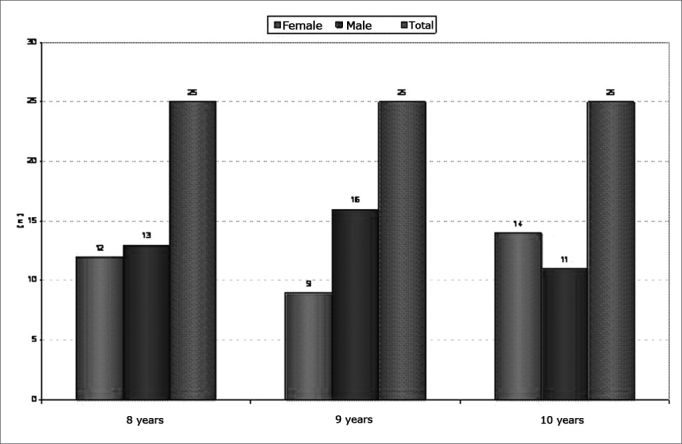
Sample characteristics according to age and male/female gender

The results of the GIN test in the sample are described below (gap-detection thresholds and percentage of correct answers) in relation to the variables ears, gender, and age. Chart 1 shows the sample characteristics according to the three age ranges and male/female gender.

Table 1 shows the mean and standard deviation values for gap-detection thresholds and the percentage of correct answers for the right and left ears, using track 1. The ear in which the test was started on track 1 was alternated: about half of the children started with the right ear and the remaining children with the left ear. The results revealed no statistically significant differences in this criterion.

**Table 1:** Mean, standard deviation, and p-value for gap-detection thresholds (in ms) and the percentage of correct answers (%) for the right and left ears for Track 1

	Test range 1						
	Right ear			Left ear			
Variable	N	mean	standard deviation	n	mean	standard deviation	p-value
Threshold	40	4,8	0,9	35	4,6	0,7	0.7105 A
Percentage of correct answers	40	73,6	6,7	35	74,6	7,4	0,5347

Student's T paired test / A Wilcoxon's paired test

Table 2 shows the analysis of track 1 compared to track 2. The statistical analysis revealed no significant difference in track 1 and 2 performance.

**Table 2:** Mean, standard deviation e p-valor for gap detection thresholds (in ms) and percentage of correct answers (%) for the Track regardless of the right or left ear

	Track 1				Track2		
Variable	N	mean	standard deviation	n	mean	standard deviation	P-value
Threshold	75	4,6	0,7	75	4,8	0,7	0.0520 A
Percentage of correct answers	75	74,0	7,0	75	73,3	7,0	0,2883

Student's T paired test / A Wilcoxon's paired test

Table 3 shows the results of comparing the mean gap-detection thresholds and percentage of correct answers for the right and left ears.

**Table 3:** Mean, standard deviation e p-valor for gap detection thresholds (in ms) and percentage of correct answers, comparing the right and left ears

	Right ear			Left ear			
Variable	n	mean	standard deviation	n	mean	standard deviation	P-value
Threshold	75	4,7	0,7	75	4,4	0,7	0.3932 A
Percentage of correct answers	75	73,2	6,7	75	74,1	7,3	0,2162

Student's T paired test / A Wilcoxon's paired test

Table 4 shows the results of the mean gap-detection thresholds and percentage of correct answers according to gender, and the statistical analysis when adding the right and left ears. There was a statistically significant difference between males and females only in the percentage of correct answers.

**Table 4:** Mean, standard deviation, median and p-value gap-detection thresholds (in ms) and percentage of correct answers, according to gender

	Female			Male			
Variable	n	mean	standard deviation	n	mean	standard deviation	P-value
Threshold	70	4,8	0,7	80	4,6	0,7	0.0740 A
Percentage of correct answers	70	72,4	7,5	80	74,7	6,4	0,0488*

Student's T paired test / A Wilcoxon's paired test

The gap-detection thresholds were analyzed for groups I, II, and III regardless of sex. Only the percentage of correct answers was analyzed for the three groups; gender was taken into account because of the male-female difference shown on Table 4.

Table 5 shows the results according to age.

**Table 5:** Mean, standard deviation e p-valor dos gap-detection thresholds (in ms) and percentage of correct answers, relative to the age range

		Group I			Group II			Group III		
Variable	n	mean	standard deviation	N	mean	standard deviation	n	mean	standard deviation	P-value
Threshold	50	4,8	0,8	50	4,6	0,7	50	4,7	0,6	0.6132 A
Percentage of correct answers										
Female	24	72,9	7,6	18	74,8	6,3	28	70,5	7,8	0,1559
Male	26	74,2	7,2	32	75,9	5,8	22	73,5	6,4	0,3511

ANOVA / A ANOVA - Kruskal-Wallis

Table 6 shows the gap-detection thresholds and percentage of correct answers independently from ears, gender and age.

**Table 6:** Description of gap detection threshold means and standard deviation (in ms) and the percentage of correct answers regardless of the ear, gender and age

	General			
Variable	n	mean	standard deviation	confidence interval
Threshold	150	4,7	0,7	6,1
Percentage of correct answers	150	73,6	7,0	60

## DISCUSSION

Studies have suggested an important correlation between abnormalities in the temporal resolution ability and some speech perception disorders and disabled reading in adults and children. Perception abilities of speech, language and reading depend deeply on temporal processing of sound, which may be assessed using the GIN test.^4^, ^18^, ^26^

The GIN test results in our study showed a similar performance in test tracks 1 and 2 regardless of which ear started the exam (we alternated the starting ear); the performance was similar between track 1 relative to track 2 (Tables 1 and 2). These results demonstrate no learning effect or fatigue, as have been observed in other similar studies.^4^, ^21^

No advantage of one ear over the other in gap-detection thresholds and percentage of correct answers were observed (Table 3). Published papers have stated that monotic tests are useful for detecting - but not locating - auditory pathway abnormalities; ipsi- and contralateral pathways are involved, resulting in a similar performance in both ears in this task.^27^ Our results concur with other published papers where no perceptual asymmetry between ears was reported for gap detection.^21^, ^28^, ^29^ Shinn et al.^30^ recently published comparable results and suggested that the auditory system maturation of temporal resolution abilities occurs similarly in both ears.

Other studies have pointed to an advantage in the right ear in temporal resolution tasks; these studies, however, applied other parameters and criteria. Brown et al.^31^ and Sulakhe et al.^32^ used the reaction time to the presence of gaps in their analysis, and noted an advantage in favor of the right ear over the left. This parameter was not investigated in our study or in other papers cited above, all of which found no right ear advantage. It is therefore evident that the same test parameters for assessing temporal resolution should be applied so that results may be debated and compared.

Males performed slightly better in the mean percentage of correct answers compared to females (72.4% x 74.7%, p=0.4888). This difference was not encountered in the mean gap-detection threshold means (Table 4). Although few studies have related gap-detection threshold test results with gender, our findings concur with other published results in Brazilian adults.^4^

An age-related comparison of results revealed no statistically significant differences among the three groups (Table 5). Other papers have reported similar results,^8^, ^18^, ^28^, ^30^ suggesting that the temporal resolution ability develops by age 7 years.^30^

Muniz et al.^18^ associated these results with the fact that the type of stimulus may alter auditory processing performance, and what is observed with [verbal] speech sounds may not occur with non-verbal sounds; this explains the full maturation of temporal abilities based on non-verbal stimuli by age 7 years.

The mean gap-detection threshold value, independently from the variables sex and age, was 4.7 ms. The total percentage of correct answers was also analyzed independently from the variables sex and age, although there was a statistical difference in the slightly superior performance of males compared to females (Table 6). The statistical analysis revealed this difference (2.3%), which, however, is not clinically relevant. Thus, we suggest a single value (73.6%) as the percentage of correct answers regardless of the gender difference.

Our results concurred with those of Musiek et al.,^28^ and Samelli and Schochat.^4^ the Brazilian authors suggested that minor differences in values are probably due to acoustic differences between English and Portuguese.

Although the GIN test used non-verbal segments, the manner in which these stimuli are processed by speakers of different languages may vary because of the specific phonetic features of each language.^33^ The acoustic and prosodic features of English and Portuguese differ in several aspects, and the temporal resolution processes required for decoding the phonemes may be different because of the demands of each language; this may result in a smaller or larger gap-detection threshold.^34^

Evidence suggests that languages such as English, which has more phonemes that are differentiated by frequency variations, produce speakers with more sensitive acoustic perception of this aspect compared to speakers of Portuguese, in which there are more phonemes that are differentiated by duration.^33^ In Portuguese, phonemes are discriminated more easily in relation to their duration compared to English; the auditory system makes less effort in this task, which could result in lower discrimination for frequency and duration tasks.^35^

Recent studies of the GIN test have yielded gap-detection thresholds with minor value differences; the values for Portuguese-speaking children were slightly superior, as in our study compared to other published results.^28^, ^30^

These results suggest that although Portuguese speakers perform worse in frequency and duration discrimination tasks, the fact that this language uses the parameter duration to discriminate phonemes may explain the better thresholds, as the auditory system requires less effort because the speaker is more familiar with a common parameter in his or her native language. Still, additional studies relating the acoustic and phonetic differences in each language are required to establish the relationship among these acoustic specificities and the temporal resolution ability.

Our results indicated lower thresholds, compared with Balen et al.'s recent study.^21^ Although the test parameters were similar, that study included 19 children, of which 10 underwent the GIN test. In addition to the small sample, it included children aged 6 to 14 years. Because of recent debates about maturation of temporal resolution by age 7 years, it is possible that children aged 6 to 7 years had higher thresholds compared to those in higher age groups, thus explaining the difference in theirs and our results.

Often it is a combination of data from several clinical tools that helps improve the efficacy of a test for evaluating any given ability and/or function.^28^ This is the case in studies - such as this study - aiming to normatize a new test, such as the GIN test. Thus, although our data are insufficient for normatizing the GIN test, given the size of the sample, the authors suggest using the 95% confidence interval as a cutoff point for normalcy, as adopted in the literature: 6,1ms (2 standard deviations plus the gap-detection threshold) and 60% (2 standard deviations below the value of the percentage of correct answers).

Based on our results and on studies about the maturation process of temporal resolution by age 7 years, we recommend further studies to evaluate this ability in children aged below 7 years, and studies with larger samples. This would add to knowledge in this are, facilitate comparisons between studies, and support the definition of normalcy criteria. This would open the possibility of additional studies of language, speech and learning disabled children, and verify the presence or absence of altered gap-detection thresholds in this population.

Having detected a disability in temporal resolution, it may be trained to improve auditory abilities, thereby supporting the phonoaudiological rehabilitation process for overcoming impaired learning.

The GIN test should be applied with extreme care in children; the same applies to an overall assessment of auditory processing, since factors such as attention, memory, motivation and fatigue may alter the results.

## CONCLUSION

There were no statistically significant differences relative to the right and left ears and age. Male subjects had a slightly superior performance only in the percentage of correct answers test, compared to female subjects. The following values were encountered based on the 95% confidence interval as a cutoff point for normality: gap-detection threshold - 6.1 ms; and percentage of correct answers - 60%.
